# Enhanced differentiation of human pluripotent stem cells into pancreatic endocrine cells in 3D culture by inhibition of focal adhesion kinase

**DOI:** 10.1186/s13287-020-02003-z

**Published:** 2020-11-16

**Authors:** Xiaofang Liu, Jinhua Qin, Mingyang Chang, Shuyong Wang, Yali Li, Xuetao Pei, Yunfang Wang

**Affiliations:** 1 Department of Obstetrics and Gynecology, Air Force Medical Center, PLA, Beijing, 100142 China; 2 Stem Cells and Regenerative Medicine Lab, Institute of Health Service and Transfusion Medicine, Beijing, 100850 China; 3 Experimental Hematology and Biochemistry Lab, Beijing Institute of Radiation Medicine, Beijing, 100850 China; 4 Army Tuberculosis Prevention and Control Key Laboratory, Beijing Key Laboratory of New Techniques of Tuberculosis Diagnosis and Treatment, Institute of Tuberculosis Research, The 8th Medical Center of Chinese PLA General Hospital, Beijing, 100091 China; 5 Department of Obstetrics and Gynecology, Chinese PLA General Hospital, Beijing, 100850 China; 6 Hepatal-Biliary-Pancreatic Center, Translational Research Center, Beijing Tsinghua Chang Gung Hospital, Beijing, 102218 China

**Keywords:** hPSCs, β cells, Three-dimensional culture, Differentiation, FAK, Connexin 36

## Abstract

**Background:**

Generation of insulin-producing cells from human pluripotent stem cells (hPSCs) in vitro would be useful for drug discovery and cell therapy in diabetes. Three-dimensional (3D) culture is important for the acquisition of mature insulin-producing cells from hPSCs, but the mechanism by which it promotes β cell maturation is poorly understood.

**Methods:**

We established a stepwise method to induce high-efficiency differentiation of human embryonic stem cells (hESCs) into mature monohormonal pancreatic endocrine cells (PECs), with the last maturation stage in 3D culture. To comprehensively compare two-dimensional (2D) and 3D cultures, we examined gene expression, pancreas-specific markers, and functional characteristics in 2D culture-induced PECs and 3D culture-induced PECs. The mechanisms were considered from the perspectives of cell–cell and cell–extracellular matrix interactions which are fundamentally different between 2D and 3D cultures.

**Results:**

The expression of the pancreatic endocrine-specific transcription factors PDX1, NKX6.1, NGN3, ISL1, and PAX6 and the hormones INS, GCG, and SST was significantly increased in 3D culture-induced PECs. 3D culture yielded monohormonal endocrine cells, while 2D culture-induced PECs co-expressed INS and GCG or INS and SST or even expressed all three hormones. We found that focal adhesion kinase (FAK) phosphorylation was significantly downregulated in 3D culture-induced PECs, and treatment with the selective FAK inhibitor PF-228 improved the expression of β cell-specific transcription factors in 2D culture-induced PECs. We further demonstrated that 3D culture may promote endocrine commitment by limiting FAK-dependent activation of the SMAD2/3 pathway. Moreover, the expression of the gap junction protein Connexin 36 was much higher in 3D culture-induced PECs than in 2D culture-induced PECs, and inhibition of the FAK pathway in 2D culture increased Connexin 36 expression.

**Conclusion:**

We developed a strategy to induce differentiation of monohormonal mature PECs from hPSCs and found limited FAK-dependent activation of the SMAD2/3 pathway and unregulated expression of Connexin 36 in 3D culture-induced PECs. This study has important implications for the generation of mature, functional β cells for drug discovery and cell transplantation therapy for diabetes and sheds new light on the signaling events that regulate endocrine specification.

**Supplementary Information:**

The online version contains supplementary material available at 10.1186/s13287-020-02003-z.

## Background

Diabetes is a globally widespread disease characterized by hyperglycemia due to autoimmune destruction of insulin (INS)-producing β cells (type 1 diabetes; T1D) or to extensive β cell exhaustion and depletion after hypersecretion of INS to overcome INS resistance (type 2 diabetes; T2D). All T1D and many T2D patients require exogenous INS delivery, and the challenges associated with managing INS dosing may lead to poor overall glycemic control. Whole pancreas or pancreatic islet (~ 6–10 × 10^5^ islets or ~ 10^9^ β cells) transplantation is considered to be one of the most effective therapies for patients with severe diabetes that does not involve exogenous INS [1–3]. However, it is severely limited by the shortage of donor organs and the necessity of life-long use of immunosuppressive drugs to prevent rejection of the transplanted islets. Human pluripotent stem cells (hPSCs), including human embryonic stem cells (hESCs) and induced pluripotent stem cells (iPSCs), can serve as renewable sources of β cells due to their capacity for extensive expansion and commitment to various somatic cell fates.

Stepwise protocols have been reported for differentiation of hPSCs into INS-secreting cells that mimic pancreatic development through the definitive endoderm (DE), primitive gut tube (PGT), pancreatic progenitors (PPs), and endocrine precursor (EP) stages, with ultimate maturation into pancreatic endocrine cells (PECs) [4–9]. Cell–cell and cell–extracellular matrix (ECM) interactions play vital roles in cell proliferation, differentiation, and functional maintenance. Pancreatic islets are three-dimensional arrangements of cells with intricate cell–cell and cell–ECM interactions. It is important that the culture environment takes into account the spatial organization of the cell. Three-dimensional (3D) cell culture more accurately imitates the in vivo conditions than traditional two-dimensional (2D) culture, as it allows cells to grow or interact with their surroundings in all three dimensions [10, 11]. Cell–cell and cell–ECM interactions have been confirmed to be essentially different between 2D and 3D cultures. These differences result in alterations of the molecular pathways that regulate cell behaviors, leading to distinct biological outcomes, such as cell phenotypes and functions [12]. A typical and clinically relevant example of a dimensionality-mediated cell response was reported in 1990 [13]. When growing as monolayers, murine mammary tumor cells did not display the drug-resistant phenotypes that previously had been seen only in vivo, while cells cultured under 3D conditions exhibited the drug resistance properties. Recently, 3D cell culture has been increasingly used for stem cell research, in which cell phenotypes need to be strictly controlled [14, 15].

Studies on islet function have found that intact islets isolated from the body have better INS release function than dispersed islet cells and that when the dispersed islet cells re-aggregated, the INS-secreting activity can be restored [16]. Bergsten et al. reported that aggregated mouse insulinoma-derived MIN6 cells, which display characteristics of pancreatic β cells, secrete INS in response to glucose stimulation [17]. These findings suggest that the spherical structure of islets may be associated with the differentiation and maturation of islet cells. It has also previously been reported that when stem cells are differentiated into INS-secreting cells, the cells spontaneously aggregated into clusters, and 3D aggregate formation is necessary to generate INS-producing cells [18]. Moreover, Suemori et al. found that 3D culture plays an important role in the induction of functional INS-expressing cells from hPSCs [19]. Although many studies have reported that 3D culture is important for the acquisition of mature INS-producing cells from hPSCs [19–21], none of them has thoroughly compared 2D and 3D cultures, and the mechanism by which 3D culture promotes β cell maturation is poorly understood.

In this study, we developed a stepwise strategy to differentiate hESCs into mature monohormonal PECs using 3D culture at the maturation stage. To comprehensively compare 2D culture and 3D culture, we examined the gene expression, pancreas-specific markers, and functional characteristics of 2D culture-induced PECs (PECs-2D) and 3D culture-induced PECs (PECs-3D). 3D culture significantly increased the pancreatic specification efficiency and enhanced the functional maturation of PECs. Furthermore, the mechanisms were considered from the perspectives of cell–cell and cell–ECM interactions, which are fundamentally different between 2D and 3D cultures. We found that 3D culture promoted endocrine commitment by limiting focal adhesion kinase (FAK)-dependent activation of the SMAD2/3 pathway and enhanced functional maturation of INS-producing cells by upregulating Connexin 36 (Cx36) expression.

## Methods

### Cell culture

The hESC line H9 (WiCell, USA) was grown in feeder-free conditions in six-well Nunclon surface plates (Nunc, USA) coated with Matrigel (R&D systems, USA) and maintained in mTESR1 media (Stem Cell Technologies, USA). Cells were passaged at a 1:3~4 ratio using dispase (Invitrogen, USA). All Matrigel plates were coated with a 1:80 dilution in Advanced DMEM-F12 (Gibco, USA) and incubated at room temperature for at least 1 h before use.

### Generation of pancreas endocrine cells (PECs) from hESCs

Human ESCs were passaged with Accutase (Sigma, USA) and plated at a density of 100,000 cells/cm^2^ in mTeSR1 media with 10 μM Y27632 (Selleckchem, USA) on RPMI1640 (Gibco, USA), Matrigel (R&D systems, USA), and collagen IV (R&D systems, USA) (5:2:1) mixed gel coated-plate (Corning, USA). In the restriction of definitive endoderm (DE) stage (S1), cells were cultured for 24 h in RPMI1640 with B-27 supplement (1:50, Gibco, USA), N-2 supplement (1:50, Gibco, USA), 100 ng/ml Activin A (R&D systems, USA), and 50 ng/ml Wnt3a (R&D systems, USA), and then treated with 100 ng/ml Activin A and 0.2% FBS (Gibco, USA) for 2 days. In the stage (S2) to get the primitive gut tube (PGT), the culture medium was replaced with RPMI1640 supplemented with B27 supplement (1:50), N2 supplement (1:50), 30 ng/ml FGF7, 5 ng/ml Wnt3a, 0.75 μM Dorsomophin (Sigma, USA), and 2% FBS (Gibco, USA) for 3 days. And in the stage (S3) of pancreatic progenitors (PPs), cells were cultured in advanced DMEM-F12 supplemented with B27 supplement (1:100), 2 μM retinoic acid (Sigma, USA), 0.25 μM cyclopamine (Selleckchem, USA), 30 ng/ml FGF7 (R&D systems, USA), 50 ng/ml Noggin (R&D systems, USA), 0.3 μM IL-5 (R&D systems, USA), and 6 μM SB431542 (Selleckchem, USA) for 3 days. At the end of stage 3, media were changed to DMEM (Gibco, USA) supplemented with B27 supplement (1:100), 50 ng/ml Exendin-4 (R&D systems, USA), 6 μM SB431542, 50 ng/ml Noggin, and 10 mM nicotinamide (Sigma, USA). For 3D culture, cells at stage 3 were digested with Accumax and replated at a density of 3 × 10^5^/ml in ultra-low attachment 6-well plates (Corning, USA), and the plates were placed on a 3D orbital shaker set at a rotation rate of 80 rpm in a 37 °C incubator, 5% CO_2_. Cells were photographed during differentiation using a Nikon Eclipse Ti-S phase-contrast microscope (Nikon, Japan).

### Quantitative real-time PCR analyses

Total RNA was isolated using an RNeasy extraction kit. RNA was reverse transcribed using Superscript II reverse transcriptase (Invitrogen, USA) according to the manufacturer’s instructions. Quantitative real-time PCR (qRT-PCR) was performed with SYBR Green real-time PCR master mix (TOYOBO, Japan) on a Bio-Rad iQ5 Real-Time PCR detection system (Bio-Rad, USA). The data were analyzed using the delta–delta Ct method. The primers are listed in supplementary Table S1.

### Immunofluorescent staining

Cells were fixed with 4% paraformaldehyde for 20 min at room temperature and blocked with 10% normal goat serum (ZSGB Biotech, China) or normal donkey serum (Abcam, USA) for 1 h, followed by incubation with primary antibodies at 4 °C overnight. Labeled isotype-specific secondary antibodies were added and incubated 1 h at room temperature. Cells were counterstained with 4′,6-diamidino-2-phenylindole (DAPI) for visualization of cell nuclei and observed using a Zeiss LSM 510 confocal microscopy (Zeiss, German) and the Zeiss LSM Image Browser Software (Zeiss, German). Antibodies used in this study are summarized in supplementary Table S2.

### Flow cytometry

Single-cell suspensions were obtained by dissociation with Accutase for 3–5 min. Cell surface antigen staining was performed in PBS at 4 °C. Intracellular staining was performed with the BD Cytofix/Cytoperm™ Kit (BD Biosciences, USA) according to the manufacturer’s instructions. Briefly, cells were fixed and permeabilized with BD Cytofix/Cytoperm solution for 20 min at 4 °C. Intracellular antigen staining was performed in BD Perm/Wash solution. The stained cells were analyzed with BD FACSAria (BD Biosciences, USA), and the data was analyzed using the Flowjo software version 10 (TreeStar, USA). The sources and concentrations of primary and secondary antibodies and isotype controls are listed in supplementary Table S2.

### Dithizone staining

The dithizone stock solution was prepared by adding 3 ml ethanol and 50 μl concentrated ammonium hydroxide to 50 mg dithizone (Sigma, USA). The clear dark-red solution was then diluted with PBS up to 30 ml and stored at − 20 °C. Next, the stock solution was diluted 1:20 in PBS. Cells were washed with PBS for three times and incubated in working solution for 10 min at 37 °C. Finally, cells were examined under a Nikon Eclipse Ti-S microscope (Nikon, Japan).

### C-peptide release assay

The pancreatic endocrine cells were used for the C-peptide release assay as previously described. Briefly, after a 1-h wash in KRBH medium, 300 μl of basal media that contain 2 mM d-glucose (Sigma) was added to each well of 12-well dishes. After 1-h incubation, the basal media were changed into 300 μl of stimulation media (20 mM d-glucose, 30 mM KCl, or 30 μM Forskolin). The cultures were incubated at 37 °C in a 5% CO_2_ environment for 30 min. For each experiment, 6 wells of supernatants were pooled together and stored at − 20 °C until assay; meanwhile, the cells were harvested for protein determination using the Bio-Rad Protein Assay K (Bio-Rad, USA) according to the Bradford method. Ultra-sensitive human C-peptide ELISA kit (Mercodia, Sweden) has been used, and the assays are done according to the manufacturer’s instructions.

### Transmission electron microscopy (TEM)

The cell samples were rinsed with PBS and fixed in 3% glutaraldehyde/0.1 M sodium cacodylate, pH 7.4 overnight. Following three rinses with sodium cacodylate buffer, the samples were postfixed for 1 h in 1% osmium tetroxide/0.1 sodium cacodylate buffer. After rinsing in deionized water, samples were dehydrated and embedded in Polybed 812 epoxy resin (Polysciences, Inc., USA). The samples were sectioned perpendicular to the substrate at 70 nm using a diamond knife. Ultrathin sections were collected on 200 mesh copper grids and stained with 4% aqueous uranyl acetate for 15 min, followed by Reynolds’ lead citrate for 7 min. Samples and stained sections were observed using a H7650 transmission electron microscope (HITACHI, Japan) operating at 80 kV (H7650 Electron Microscopy) and photographed using an AMT XR16M CCD Digital Camera and AMT Capture Engine Software version 600.259 (Advanced Microscopy Techniques Corp, USA).

### Western blotting

Cells were harvested in lysis buffer (50 mM Tris-HCl, pH 7.4, 0.25 mM sodium deoxycholate, 150 mM NaCl, 2 mM EDTA, 0.1% sodium dodecyl sulfate, 1% Triton X-100) containing protease and phosphatase inhibitors (Roche, USA). Lysates were sonicated for 30 s, maintained on ice for 30 min, and then spun at 15,000 rpm for 15 min at 4 °C. Proteins were separated by sodium dodecyl sulfate–polyacrylamide gel electrophoresis, transferred to polyvinylidene difluoride membranes, and probed with antibodies listed in supplementary Table S2. Proteins were detected by enhanced chemiluminescence HRP substrate (Millipore, USA).

### CCK-8 assay

Cell proliferation was assessed by Cell Counting Kit-8 (Dojindo, Japan) assay. PPs from stage 3 were seeded at 2000 cells/well into 96-well plates with 100-μl culture medium and were incubated at 37 °C overnight. The 10 μl of CCK-8 solution was added to the cells at specific time points, and cells were incubated for 2 h at 37 °C. The optical density (OD) value of each well was measured using a SpectraMax M5 microplate reader (Molecular Devices, USA) at the wavelength of 450 nm.

### Statistics

Data are shown as mean ± SD. For most statistic evaluation, 2-tailed Student’s *t* test was applied for calculating statistical probability in this study. Multi-group comparisons were conducted using the two-way ANOVA. *p* values less than 0.05 were considered to be statistically significant. For all statistics, data from at least three independent samples or repeated experiments were used.

## Results

### Generation of PECs from hESCs

**Fig. 1:**
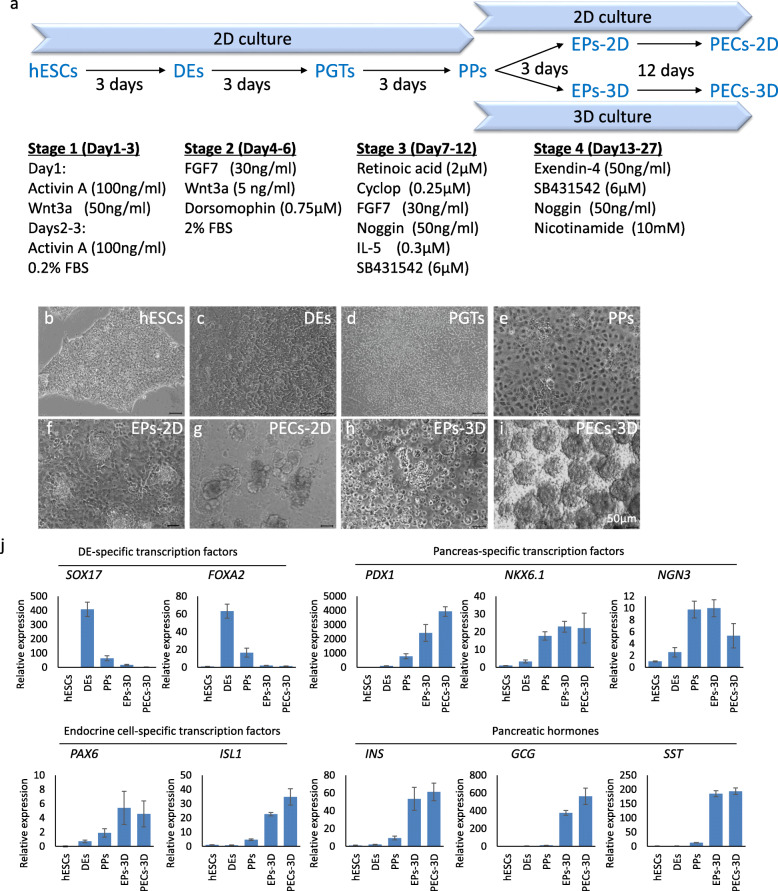
Differentiation of human embryonic stem cells to pancreatic endocrine cells. **a** Schematic overview of the protocols used for the differentiation of human embryonic stem cells (hESCs) into pancreatic endocrine cells (PECs). The hESCs are differentiated through the stages of definitive endoderm (DEs), primitive gut tube (PGTs), pancreatic progenitors (PPs), and endocrine precursors (EPs) to yield PECs using a 4-stage protocol. In stage 4, PPs were either cultured in 2D to produce EPs-2D and PECs-2D or transferred to 3D culture for differentiation into EPs-3D and PECs-3D. **b**–**i** Representative morphology of hESCs (**b**), DEs (**c**), PGTs (**d**), PPs (**e**), EPs-2D (**f**), PECs-2D (**g**), EPs-3D (**h**), and PECs-3D (**i**). Scale bars, 50 μm. **j** qRT-PCR analysis of DE-specific transcription factors (*SOX17* and *FOXA2*), pancreas-specific transcriptional factors (*PDX1*, *NKX6.1*, and *NGN3*), endocrine cell-specific transcription factors (*PAX6* and *ISL1*), and pancreatic hormones (*INS*, *GCG*, and *SST*) in hESCs, DEs, PPs, EPs-3D, and PECs-3D

Our strategy to induce PECs from hESCs in vitro is outlined in Fig. 1a. A stepwise four-stage protocol modified from the methods of previous studies [4, 6] was used to induce hESC differentiation through the stages of DEs, PGTs, PPs, and EPs stages to yield PECs, with the first three stages in monolayer 2D culture and the last stage in 2D or 3D culture (Fig. 1b–i). Sex determining region Y (SRY)-box 17 (SOX17)- and forkhead box protein A2 (FOXA2)-positive DE was efficiently induced in stage 1, with high expression levels of EpCAM and CXCR4 (Fig. 1j and Figure S1). The pancreas-specific transcription factors pancreatic and duodenal homeobox 1 (PDX1), NK6 homeobox transcription factor-related locus 1 (NKX6.1), and Neurogenin 3 (NGN3) were significantly upregulated in PPs; over 95% of PPs co-expressed PDX1 and NKX6.1 (Fig. 1j and Figure S1). In addition, flow cytometry analysis showed that more than 68% of PPs expressed CD142, a surface marker used for enrichment of pancreatic endoderm cells; this percentage is much higher than previously reported [21, 22] (Figure S2). In stage 4, when the 2D culture was continued, large numbers of cell clusters that topologically resembled normal pancreatic islets emerged from the underlying monolayer cells (Fig. 1g). To mimic pancreatic islet development, we dissociated PPs from stage 3 into single cells and replated them in ultra-low-attachment cell culture plates for 3D culture. The cells in suspension self-assembled to form three-dimensional clusters with diameters ranging from 100 to 400 μm (Fig. 1i). The endocrine cell-specific transcription factors paired box 6 (PAX6) and ISL LIM homeobox 1 (ISL1) and hormones INS, glucagon (GCG), and somatostatin (SST) were induced in EPs and PECs from 3D culture (Fig. 1j and Figure S1). Overall, hPSCs could be differentiated into PECs in a stepwise manner following our four-stage protocol.

### 3D culture promoted the maturation of PECs

**Fig. 2:**
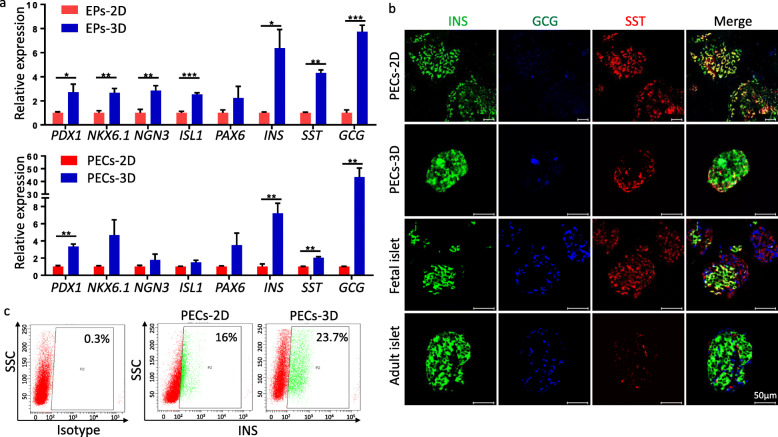
Comparison of pancreatic endocrine cells derived from 2D and 3D cultures. **a** qRT-PCR analysis of pancreas-specific transcriptional factors (*PDX1*, *NKX6.1*, and *NGN3*), endocrine cell-specific transcription factors (*PAX6* and *ISL1*), and pancreatic hormones (*INS*, *GCG*, and *SST*) in EPs-2D, EPs-3D, PECs-2D, and PECs-3D. **b** Immunostaining of insulin (INS), glucagon (GCG), and somatostatin (SST) in PECs induced under 2D or 3D culture conditions and fetal or adult islets. **c** Flow cytometry analysis of INS in PECs-2D or PECs-3D. Data represent mean ± SD (*n* = 3), *p < 0.05, **p < 0.01, ***p < 0.001

To investigate the effect of 3D culture on pancreatic differentiation, PECs induced under 2D and 3D cultures were compared at different levels. qRT-PCR analysis showed that the mRNA expression levels of transcription factors *PDX1*, *NKX6.1*, *NGN3*, *ISL1*, and *PAX6* and the hormones *INS*, *GCG*, and *SST* were significantly higher in 3D culture-induced EPs (EPs-3D) and PECs (PECs-3D) (Fig. 2a). Immunostaining of pancreatic hormones illustrated that many PECs-2D co-expressed INS and GCG or INS and SST, and some cells even expressed all three hormones, exhibiting an expression pattern resembling that of primary fetal islets (Fig. 2b). Nevertheless, the three hormones were mostly expressed in different PECs-3D; a large proportion of monohormonal INS-expressing β cells and a moderate percentage of monohormonal GCG-expressing α cells were observed. Although the percentage of SST-positive cells was slightly elevated, the hormone expression pattern in PECs-3D was most similar to that in primary adult islets (Fig. 2b). Moreover, the percentage of INS-expressing cells was much higher among PECs-3D than PECs-2D (Fig. 2c), as was also the case for GCG- and SST-expressing cells (Figure S3). Taken together, these data highlight the differences between PECs-2D and PECs-3D and suggest that 3D culture promotes endocrine cell maturation.

### Enhanced β cell function in 3D culture-induced PEC

**Fig. 3:**
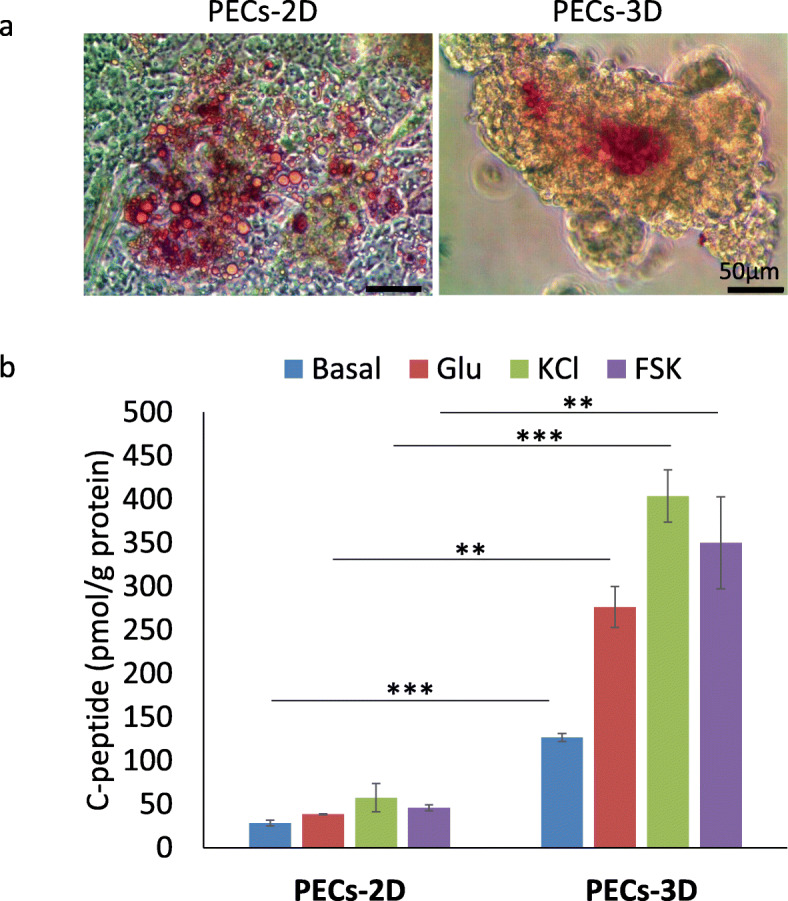
3D culture enhanced function maturation of INS-producing cells. **a** Representative images of PECs-2D and PECs-3D stained with dithizone. Scale bars, 50 μm. **b** Secreted C-peptide in response to high (20 mM) concentrations of d-glucose (Glu), 30 mM potassium chloride (KCl), or 30 μM Forskolin (FSK) was measured with a C-peptide ELISA kit. Data represent mean ± SD (*n* = 3), **p < 0.01,***p < 0.001

The β cells in islets can be specifically labeled with the zinc-chelating dye dithizone (DTZ) owing to the presence of zinc in INS-containing secretory granules [23]; therefore, DTZ can be used to efficiently stain INS-expressing regions of PECs in cultures. We observed discrete areas of DTZ staining in PECs-2D and much darker DTZ staining in PECs-3D (Fig. 3a). Analysis of ultrastructure by transmission electron microscopy (TEM) revealed that PECs-3D contained numerous endocrine granules with typical morphological characteristics of INS-containing granules (Figure S4). As previously reported, three types of INS granules were generally observed in mature human β cells by TEM: (i) granules with a light gray, diffuse core; (ii) granules with a dense, round core; and (iii) granules with a dense, rod-shaped core with a crystalline appearance [8]. Notably, we observed examples of each type of INS granule in hESC-derived PECs-3D (Figure S4).

To confirm the de novo synthesis and release of INS by hESC-derived β cells, we monitored the release of C-peptide into the culture medium in response to high glucose and stimuli (Fig. 3b). Under basal glucose conditions, PECs-3D released over four times more C-peptide than PECs-2D. Moreover, we observed a 2-fold induction of human C-peptide release from PECs-3D exposed to high glucose over the course of a 1-h incubation, while PECs-2D did not respond sensitively to high glucose (Glu). Direct depolarization of the cells via addition of potassium chloride (KCl) and activation of the cAMP signal with Forskolin (FSK) markedly increased C-peptide secretion in PECs-3D during a 1-h incubation. Together, these data suggest that PECs-3D were capable of producing appropriately packaged INS granules. Moreover, PECs-3D could respond more sensitively to glucose and stimuli than PECs-2D, which indicates that 3D culture enhances functional maturation of hESC-derived β cells.

### 3D culture might promote endocrine specification by inhibiting FAK-dependent activation of the SMAD2/3 pathway

**Fig. 4:**
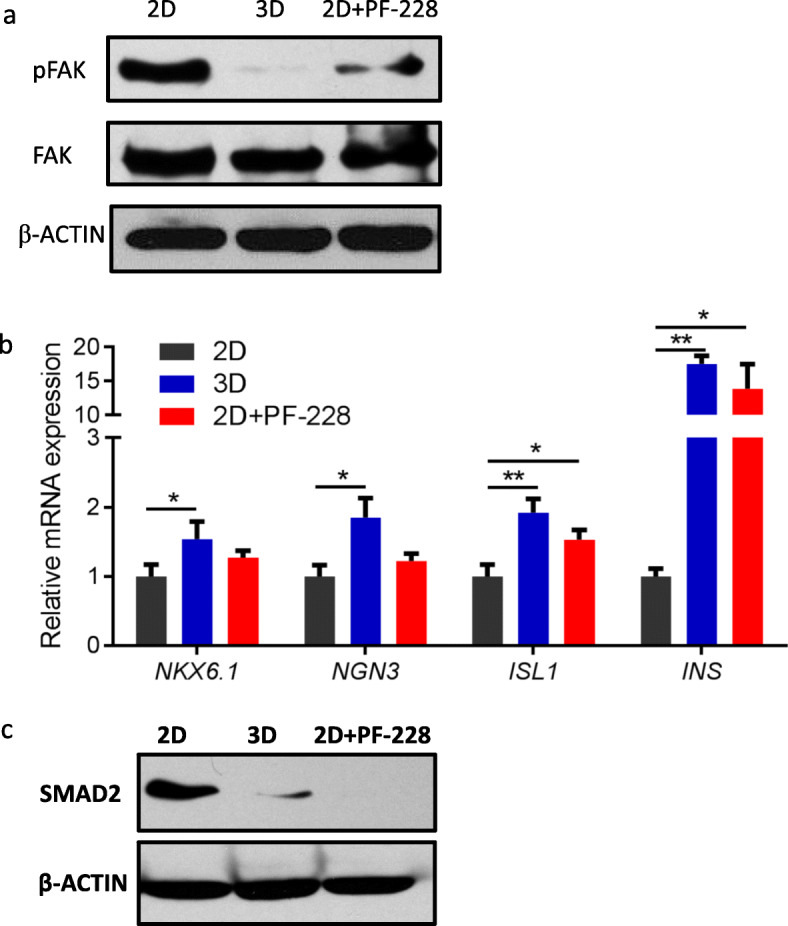
Inhibition of focal adhesion kinase (FAK) signaling and transforming growth factor-β (TGFβ) signaling was involved in 3D culture. **a** Western blot showing phosphorylated FAK (pFAK) levels in cells induced under 2D culture, 3D culture, and 2D culture treated with PF-228. At the end of stage 3, PPs were treated with (2D+PF-228) or without (2D) 2 μM PF-228 under 2D culture, or PPs were transferred to 3D culture (3D), and the lysates were collected 48 h later. The membranes were probed with pAbs specific for pFAK (Tyr-397) or total FAK. **b** qRT-PCR analysis of PECs induced under 2D culture, 3D culture, and 2D culture treated with PF-228. **c** Western blot showing SMAD2 expression levels in PECs induced under 2D culture, 3D culture, and 2D culture treated with PF-228. Data represent mean ± SD (*n* = 3), *p < 0.05, **p < 0.01

In contrast to conventional 2D monolayer culture, 3D culture is thought to mimic the natural environment found in vivo, allowing cells to interact with each other, the ECM, and their microenvironment. FAK, a central regulator of integrin signaling, that alters the association between cells and the underlying ECM, was examined in PECs-2D and PECs-3D. We noticed that phosphorylated FAK (pFAK) was nearly undetectable in PECs-3D, while pFAK levels were high in PECs-2D (Fig. 4a). Therefore, we further determined whether pharmacological inhibition of FAK would promote endocrine specification under 2D culture. PF-228 is a small molecule inhibitor that selectively inhibits FAK catalytic activity by blocking phosphorylation at Tyr-397 [24]. At the end of stage 3, PPs were treated with or without 2 μM PF-228 for 48 h under 2D culture. The proliferative ability of the cells was not affected by the treatment with PF-228, as assessed by CCK-8 assay (Figure S5). Western blot analysis showed that the FAK inhibitor PF-228 abolished the phosphorylation of FAK, as expected. Importantly, the mRNA expression of the proendocrine transcription factor *NGN3*, the β cell-specific transcription factors *NKX6.1* and *ISL1*, and *INS* were elevated in the presence of PF-228 (Fig. 4b). Together, these data suggest that the 3D culture promoted endocrine specification by inhibiting FAK activation.

Previous studies have confirmed that inhibition of SFK/FAK signaling promotes endocrine specification of human embryonic stem cell derivatives by limiting activation of the transforming growth factor-β (TGFβ)/SMAD2/3 pathway [6, 25]. We also observed a sharp decrease in SMAD2 expression in PECs-3D and confirmed that the presence of PF-228 in 2D culture significantly downregulated SMAD2 expression (Fig. 4c). Based on these observations, we concluded that 3D culture may promote endocrine commitment by limiting FAK-dependent activation of the SMAD2/3 pathway.

### 3D culture might enhance β cell function by regulating Cx36

**Fig. 5:**
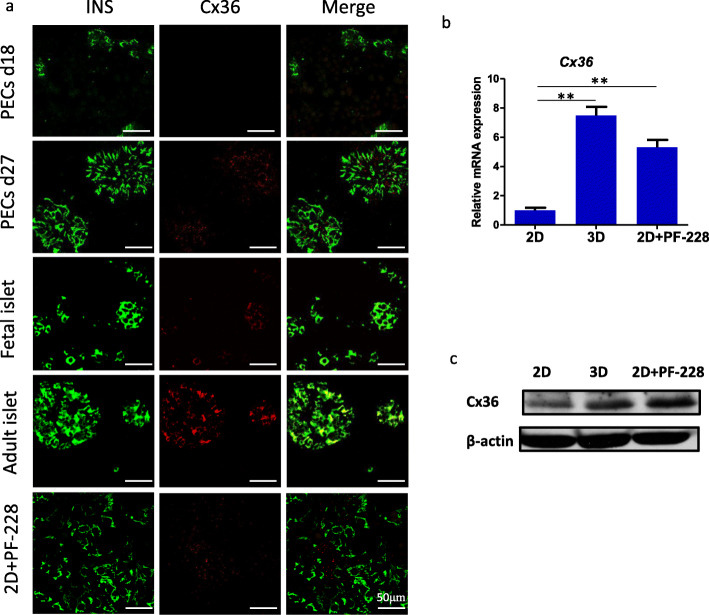
Upregulation of Cx36 in PECs induced under 3D culture. **a** Immunostaining of INS and Cx36 in hESC-derived PECs on days 18 and 27, PECs induced under 2D culture with PF-228 (2D+PF-228), fetal islets, and adult islets. Scale bar, 50 μm. **b** qRT-PCR analysis of Cx36 in PECs induced under 2D culture, 3D culture, and 2D culture treated with PF-228 (2D+PF-228). **c** Western blot showing Cx36 expression levels in PECs induced under 2D culture, 3D culture, and 2D culture treated with PF-228 (2D+PF-228). Data represent mean ± SD (*n* = 3), **p < 0.01

As described above, compared with 2D monolayer culture, 3D culture not only improved pancreatic differentiation efficiency, but also enhanced the INS secretory response to glucose. Cell–cell coupling mediated by gap junctions formed from connexin contributes to the control of INS secretion in the endocrine pancreas. INS-secreting β cells within the pancreatic islets are exclusively coupled by Cx36 gap junctions in mice and are strongly coupled by Cx36 gap junctions in humans [26]. It has been reported that adult β cells, which respond to glucose, express significantly higher levels of Cx36 than fetal β cells, which respond poorly to sugar [27, 28]. We confirmed by immunostaining that the INS-expressing cells among human adult β cells expressed much higher levels of Cx36 protein than those among human fetal β cells (Fig. 5a). And we observed that Cx36 was expressed in INS-expressing cells in the late maturation stage (day 27), but not in the early stage of differentiation, although some cells already expressed INS at the early stage (Fig. 5a). Moreover, Cx36 expression was much higher in PECs-3D than in PECs-2D, as determined by qRT-PCR and western blot analyses, suggesting that 3D culture might enhance glucose responsiveness by promoting Cx36 expression (Fig. 5b, c). Furthermore, treatment with PF-228 in 2D culture increased Cx36 expression at both the mRNA and protein levels, indicating that the FAK signaling pathway was involved in Cx36 regulation (Fig. 5b, c). Taken together, our data suggest that 3D culture might regulate Cx36 expression by inhibiting the FAK pathway, thus promoting β cell maturation.

## Discussion

Cells naturally grow, differentiate, and mature in a 3D environment. 3D cell culture models can almost perfectly mimic in vivo cell behaviors and organization; therefore, 3D culture enables accurate reproduction of these characteristics in vitro. We established a four-stage differentiation method for the induction of high-efficiency PEC differentiation from hESCs, with the first three stages in monolayer culture and the last maturation stage in 3D culture. Following this protocol, hESCs were converted to DEs, PGTs, PPs, EPs, and PECs; thus, the protocol mimicked the natural developmental events that guide the stepwise formation of mature islet cells in the pancreas.

Comparison of PECs-2D and PECs-3D showed that the expression of the pancreas-specific transcription factors *PDX1*, *NKX6.1*, *NGN3*, *ISL1*, and *PAX6* and the endocrine hormones *INS*, *GCG*, and *SST* was significantly higher in PECs-3D than in PECs-2D. Importantly, 3D culture gave rise to monohormonal endocrine cells, while PECs-2D co-expressed INS and GCG or INS and SST, and some cells even expressed all three hormones. Bruin et al. demonstrated that hESC-derived polyhormonal INS-expressing cells lacked a mechanism to import glucose, because the glucose transporter was transcribed but not translated [29]. Additionally, hESC-derived polyhormonal INS-expressing cells have been found to display only mild K^+^ channel activity that does not appear to be mediated by functional K_ATP_ channels [29–31]. Furthermore, the processing of proinsulin to form the mature INS hormone is hindered in polyhormonal INS-expressing cells as a result of a lack of the prohormone convertase expression [29, 32, 33]. The defects in glucose transporter expression, K_ATP_ channel function, and prohormone processing enzymes may contribute to the lack of glucose responsiveness in hESC-derived polyhormonal INS-producing cells. Consistent with these studies, our PECs-3D responded more sensitively to glucose and stimuli than PECs-2D. These data suggest that 3D culture promotes the functional maturation of PECs.

3D cell culture mimics the specificity of native tissue with greater physiological relevance than conventional 2D culture, because it establishes physiological cell–cell and cell–ECM interactions. Integrin receptors play major roles in tissue morphogenesis and homeostasis by regulating cell interactions with ECM proteins [34]. Furthermore, integrin receptors expressed in the human fetal pancreas play multiple roles in islet cell biological processes, including adhesion, function, and survival [35]. FAK represents a crosstalk point for integrin signaling, which is activated by integrin ligation and clustering [36]. FAK signaling alters the associations between cells and the underlying ECM, which in turn can have profound consequences for anchorage-dependent growth and differentiation [37, 38]. It has been reported that inhibition of SFK/FAK signaling potentiates endocrine differentiation by inhibiting the TGFβ/SMAD2/3 pathway [25]. Previous studies have also shown that pharmacological inhibitors that target the TGFβ type I receptor ALK5 (ALK5 inhibitor II) or ALK5 and its relatives ALK4 and ALK7 (SB431542) promote the endocrine specification of hESC derivatives [6] and the subsequent derivation of INS-producing β cells [39]. We observed lower FAK phosphorylation levels and decreased SMAD2 expression in PECs-3D than in PECs-2D. In the presence of the FAK inhibitor PF-228 in 2D culture, FAK phosphorylation was abolished, and SMAD2 expression was downregulated. Furthermore, inhibition of FAK with PF-228 in 2D culture increased the expression of *NGN3*, *NKX6.1*, *ISL1*, and *INS*, suggesting that 3D culture may promote endocrine commitment by limiting FAK-dependent activation of the SMAD2/3 pathway.

INS secretion and most other functions of pancreatic islets involve multicellular processes, which allow for rapid regulation of hormonal secretion in order to match the changing levels of circulating glucose. The INS-producing β cells of pancreatic islets are connected by a large number of small gap junction plaques, which ensure cell-to-cell coupling via Cx36 gap junctions [40]. Glucose stimulation induces much stronger secretory and metabolic responses from either intact pancreatic islets or clusters of islet cells than from single β cells [41, 42]. In our study, Cx36 expression was much higher in PECs-3D than in PECs-2D; consequently, coupling between pancreatic β cells to synchronize the activity of individual cells was better among PECs-3D than among PECs-2D. In addition, previous studies have demonstrated that changes in Cx36 alter the expression of specific β cell genes that play key roles in glucose-induced INS secretion [43, 44]. We found that treatment with PF-228 in 2D culture increased Cx36 expression. Collectively, our results indicate that 3D culture might enhance glucose responsiveness by promoting Cx36 expression, and the FAK signaling pathway is involved in Cx36 regulation.

## Conclusion

In conclusion, we developed a differentiation strategy to induce differentiation of monohormonal mature PECs from hPSCs with the last maturation step in 3D culture. In particular, 3D culture increased the differentiation efficiency and promoted the functional maturation of hESC-derived PECs. Moreover, we investigated the mechanism and found limited FAK-dependent activation of the SMAD2/3 pathway and upregulated expression of Cx36 in PECs-3D, indicating that 3D culture promoted endocrine specification of hESCs through comprehensive modulation of cell–cell and cell–ECM interactions. Our method might provide a new platform for in vitro anti-diabetic drug discovery and characterization for human metabolism and diabetes. In addition, the development of small compound inhibitors which can enhance the derivation of β cells prior to transplantation will likely help bring us closer to developing a universal cell-based therapy for diabetes.

## Supplementary Information

**Additional file 1:** **Figure S1.** Immunostaining of stage-specific markers in DEs, PPs and PECs. Scale bars, 50 μm. **Figure S2.** Flow cytometry analysis of CD142 expression in PPs. **Figure S3.** Flow cytometry analysis of Glucagon (GCG) and Somatostatin (SST) in PECs-2D and PECs-3D. **Figure S4.** TEM images of PECs-2D or PECs-3D illustrating the ultrastructure of endocrine granules. Insulin granules could be categorized into three main types: pale, diffuse gray core (open arrow); dense round core (solid arrow); dense rod-shaped core (arrowhead). Scale bars, 500 nm. **Table S1.** Primers used in this study. **Table S2.** Antibodies used in this study.

## Data Availability

The datasets supporting the conclusions of this article are included within the article and its additional files.

## Abbreviations

hPSCs: Human pluripotent stem cells
2D: Two-dimensional
3D: Three-dimensional
hESCs: Human embryonic stem cells
PECs: Pancreatic endocrine cells
DE: Definitive endoderm
PGT: Primitive gut tube
PPs: Pancreatic progenitors
EPs: Endocrine precursors
ECM: Extracellular matrix
Cx36: Connexin 36
T1D: Type 1 diabetes
T2D: Type 2 diabetes
DTZ: Dithizone
TEM: Transmission electron microscopy
FAK: Focal adhesion kinase

## References

[1] Lock LT, Tzanakakis ES (2007). Stem/progenitor cell sources of insulin-producing cells for the treatment of diabetes. Tissue Eng.

[2] Shapiro AM, Lakey JR, Ryan EA, Korbutt GS, Toth E, Warnock GL, Kneteman NM, Rajotte RV (2000). Islet transplantation in seven patients with type 1 diabetes mellitus using a glucocorticoid-free immunosuppressive regimen. N Engl J Med.

[3] Ryan EA, Paty BW, Senior PA, Bigam D, Alfadhli E, Kneteman NM, Lakey JR, Shapiro AM (2005). Five-year follow-up after clinical islet transplantation. Diabetes.

[4] D'Amour KA, Bang AG, Eliazer S, Kelly OG, Agulnick AD, Smart NG, Moorman MA, Kroon E, Carpenter MK, Baetge EE (2006). Production of pancreatic hormone–expressing endocrine cells from human embryonic stem cells. Nat Biotechnol.

[5] Jiang W, Shi Y, Zhao D, Chen S, Yong J, Zhang J, Qing T, Sun X, Zhang P, Ding M (2007). In vitro derivation of functional insulin-producing cells from human embryonic stem cells. Cell Res.

[6] Nostro MC, Sarangi F, Ogawa S, Holtzinger A, Corneo B, Li X, Micallef SJ, Park IH, Basford C, Wheeler MB (2011). Stage-specific signaling through TGF family members and WNT regulates patterning and pancreatic specification of human pluripotent stem cells. Development.

[7] Rezania A, Bruin JE, Arora P, Rubin A, Batushansky I, Asadi A, O'Dwyer S, Quiskamp N, Mojibian M, Albrecht T (2014). Reversal of diabetes with insulin-producing cells derived in vitro from human pluripotent stem cells. Nat Biotechnol..

[8] Pagliuca FW, Millman JR, Gurtler M, Segel M, Van Dervort A, Ryu JH, Peterson QP, Greiner D, Melton DA (2014). Generation of functional human pancreatic beta cells in vitro. Cell.

[9] Russ HA, Parent AV, Ringler JJ, Hennings TG, Nair GG, Shveygert M, Guo T, Puri S, Haataja L, Cirulli V (2015). Controlled induction of human pancreatic progenitors produces functional beta-like cells in vitro. EMBO J.

[10] Abbott A (2003). Biology’s new dimension. Nature.

[11] Haycock JW, Haycock JW (2011). 3D cell culture: a review of current approaches and techniques. 3D cell culture: methods and protocols.

[12] Ravi M, Paramesh V, Kaviya SR, Anuradha E, Solomon FDP (2015). 3D cell culture systems: advantages and applications. J Cell Physiol.

[13] Teicher B, Herman T, Holden S, Wang Y, Pfeffer M, Crawford J, Frei E (1990). Tumor resistance to alkylating agents conferred by mechanisms operative only in vivo. Science.

[14] Kraehenbuehl TP, Langer R, Ferreira LS (2011). Three-dimensional biomaterials for the study of human pluripotent stem cells. Nat Methods.

[15] Zujur D, Kanke K, Lichtler AC, Hojo H, Chung U-I, Ohba S (2017). Three-dimensional system enabling the maintenance and directed differentiation of pluripotent stem cells under defined conditions. Sci Adv.

[16] Berney T, Johnson PR (2010). Donor pancreata: evolving approaches to organ allocation for whole pancreas versus islet transplantation. Transplantation.

[17] Chowdhury A, Dyachok O, Tengholm A, Sandler S, Bergsten P (2013). Functional differences between aggregated and dispersed insulin-producing cells. Diabetologia.

[18] Baeyens L, Breuck S, Lardon J, Mfopou JK, Rooman I, Bouwens L (2004). In vitro generation of insulin-producing beta cells from adult exocrine pancreatic cells. Diabetologia.

[19] Takeuchi H, Nakatsuji N, Suemori H (2014). Endodermal differentiation of human pluripotent stem cells to insulin-producing cells in 3D culture. Sci Rep.

[20] Jiang J, Au M, Lu K, Eshpeter A, Korbutt G, Fisk G, Majumdar AS (2007). Generation of insulin-producing islet-like clusters from human embryonic stem cells. Stem Cells.

[21] Kelly OG, Chan MY, Martinson LA, Kadoya K, Ostertag TM, Ross KG, Richardson M, Carpenter MK, D'Amour KA, Kroon E (2011). Cell-surface markers for the isolation of pancreatic cell types derived from human embryonic stem cells. Nat Biotechnol.

[22] Ramond C, Beydag-Tasoz BS, Azad A, van de Bunt M, Petersen MBK, Beer NL, Glaser N, Berthault C, Gloyn AL, Hansson M, et al. Understanding human fetal pancreas development using subpopulation sorting, RNA sequencing and single-cell profiling. Development. 2018;145(16):dev165480. 10.1242/dev.165480 PMC6124547 30042179

[23] A simple method of staining fresh and cultured islets. Transplantation 1988, 45:827–829. 2451869

[24] Slack-Davis JK, Martin KH, Tilghman RW, Iwanicki M, Ung EJ, Autry C, Luzzio MJ, Cooper B, Kath JC, Roberts WG, Parsons JT (2007). Cellular characterization of a novel focal adhesion kinase inhibitor. J Biol Chem.

[25] Afrikanova I, Yebra M, Simpkinson M, Xu Y, Hayek A, Montgomery A (2011). Inhibitors of Src and focal adhesion kinase promote endocrine specification: impact on the derivation of beta-cells from human pluripotent stem cells. J Biol Chem.

[26] Farnsworth NL, Benninger RKP (2014). New insights into the role of connexins in pancreatic islet function and diabetes. FEBS Lett.

[27] Navarro-Tableros V, Fiordelisio T, Hernández-Cruz A, Hiriart M (2007). Physiological development of insulin secretion, calcium channels, and GLUT2 expression of pancreatic rat β-cells. Am J Physiol Endocrinol Metab.

[28] Carvalho CP, Barbosa HC, Britan A, Santos-Silva JC, Boschero AC, Meda P, Collares-Buzato CB (2010). Beta cell coupling and connexin expression change during the functional maturation of rat pancreatic islets. Diabetologia.

[29] Bruin JE, Erener S, Vela J, Hu X, Johnson JD, Kurata HT, Lynn FC, Piret JM, Asadi A, Rezania A, Kieffer TJ (2014). Characterization of polyhormonal insulin-producing cells derived in vitro from human embryonic stem cells. Stem Cell Res.

[30] Basford CL, Prentice KJ, Hardy AB, Sarangi F, Micallef SJ, Li X, Guo Q, Elefanty AG, Stanley EG, Keller G (2012). The functional and molecular characterisation of human embryonic stem cell-derived insulin-positive cells compared with adult pancreatic beta cells. Diabetologia.

[31] Li C, Ackermann AM, Boodhansingh KE, Bhatti TR, Liu C, Schug J, Doliba N, Han B, Cosgrove KE, Banerjee I (2017). Functional and metabolomic consequences of KATP channel inactivation in human islets. Diabetes.

[32] Fukayama M, Ogawa M, Hayashi Y, Koike M (1986). Development of human pancreas: immunohistochemical study of fetal pancreatic secretory proteins. Differentiation.

[33] Zhu X, Orci L, Carroll R, Norrbom C, Ravazzola M, Steiner DF (2002). Severe block in processing of proinsulin to insulin accompanied by elevation of des-64,65 proinsulin intermediates in islets of mice lacking prohormone convertase 1/3. Proc Natl Acad Sci.

[34] Alam N, Goel HL, Zarif MJ, Butterfield JE, Perkins HM, Sansoucy BG, Sawyer TK, Languino LR (2007). The integrin—growth factor receptor duet. J Cell Physiol.

[35] Wang R, Li J, Lyte K, Yashpal NK, Fellows F, Goodyer CG (2005). Role for β1 integrin and its associated α3, α5, and α6 subunits in development of the human fetal pancreas. Diabetes.

[36] Parsons JT (2003). Focal adhesion kinase: the first ten years. J Cell Sci.

[37] Pala D, Kapoor M, Woods A, Kennedy L, Liu S, Chen S, Bursell L, Lyons KM, Carter DE, Beier F, Leask A (2008). Focal adhesion kinase/Src suppresses early chondrogenesis. J Biol Chem.

[38] Bursell L, Woods A, James CG, Pala D, Leask A, Beier F (2007). Src kinase inhibition promotes the chondrocyte phenotype. Arthritis Res Ther.

[39] Rezania A, Bruin JE, Riedel MJ, Mojibian M, Asadi A, Xu J, Gauvin R, Narayan K, Karanu F, O’Neil JJ (2012). Maturation of human embryonic stem cell-derived pancreatic progenitors into functional islets capable of treating pre-existing diabetes in mice. Diabetes.

[40] Serre-Beinier V, Le Gurun S, Belluardo N, Trovato-Salinaro A, Charollais A, Haefliger JA, Condorelli DF, Meda P (2000). Cx36 preferentially connects beta-cells within pancreatic islets. Diabetes.

[41] Salomon D, Meda P (1986). Heterogeneity and contact-dependent regulation of hormone secretion by individual B cells. Exp Cell Res.

[42] Bosco D, Meda P (1991). Actively synthesizing beta-cells secrete preferentially after glucose stimulation. Endocrinology.

[43] Pérez-Armendariz EM (2013). Connexin 36, a key element in pancreatic beta cell function. Neuropharmacology.

[44] Le Gurun S, Martin D, Formenton A, Maechler P, Caille D, Waeber G, Meda P, Haefliger J-A (2003). Connexin-36 contributes to control function of insulin-producing cells. J Biol Chem.

