# Supplementary material for: Enhanced differentiation of human pluripotent stem cells into pancreatic endocrine cells in 3D culture by inhibition of focal adhesion kinase
Source: Stem Cell Res Ther. 2020 Nov 16;11:488. doi: 10.1186/s13287-020-02003-z (PMC7667734; doi:10.1186/s13287-020-02003-z)
Supplement: Supplementary file 1 — Additional file 1: Figure S1. Immunostaining of stage-specific markers in DEs, PPs and PECs. Scale bars, 50 μm. Figure S2. Flow cytometry analysis of CD142 expression in PPs. Figure S3. Flow cytometry analysis of Glucagon (GCG) and Somatostatin (SST) in PECs-2D and PECs-3D. Figure S4. TEM images of PECs-2D or PECs-3D illustrating the ultrastructure of endocrine granules. Insulin granules could be categorized into three main types: pale, diffuse gray core (open arrow); dense round core (solid arrow); dense rod-shaped core (arrowhead). Scale bars, 500 nm. Table S1. Primers used in this study. Table S2. Antibodies used in this study. [file 13287_2020_2003_MOESM1_ESM.pdf]

Supplementary figures and tables

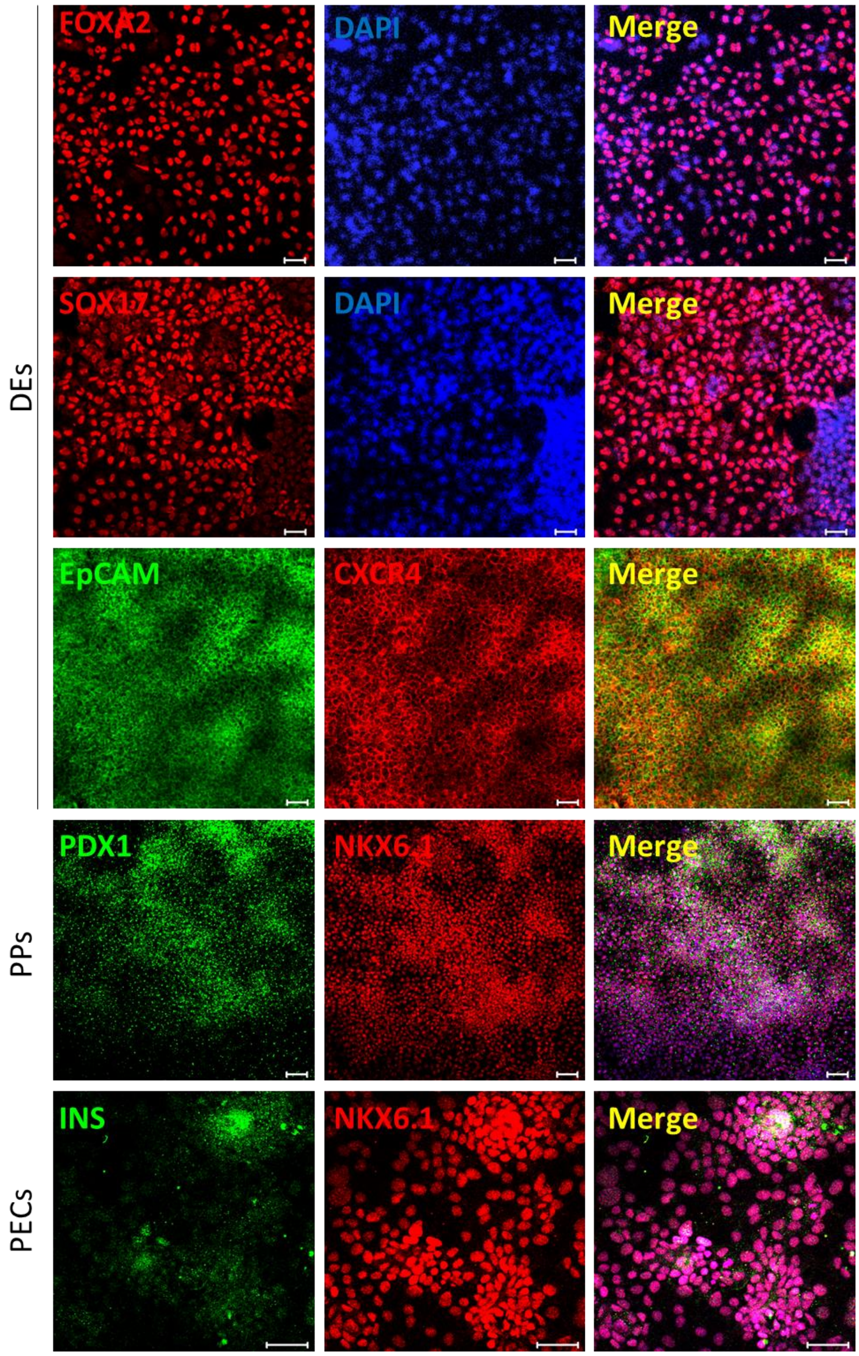

Figure S1. Immunostaining of stage-specific markers in DEs, PPs and PECs. Scale bars, 50 μm.

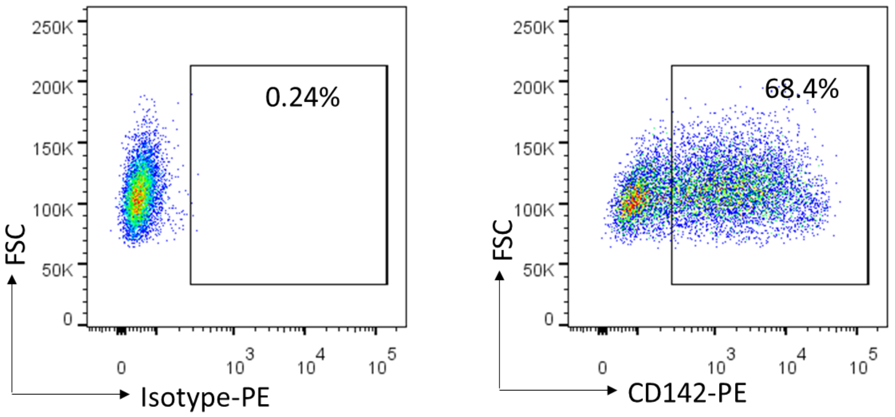

Figure S2. Flow cytometry analysis of CD142 expression in PPs.

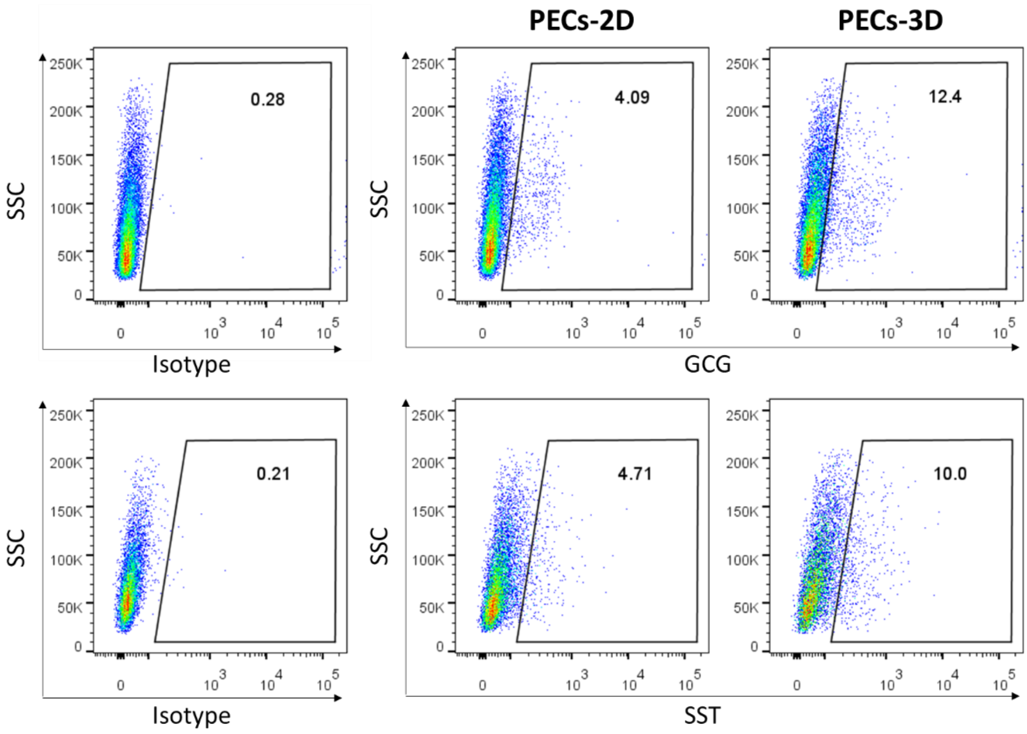

Figure S3. Flow cytometry analysis of Glucagon (GCG) and Somatostatin (SST) in PECs-2D and PECs-3D.

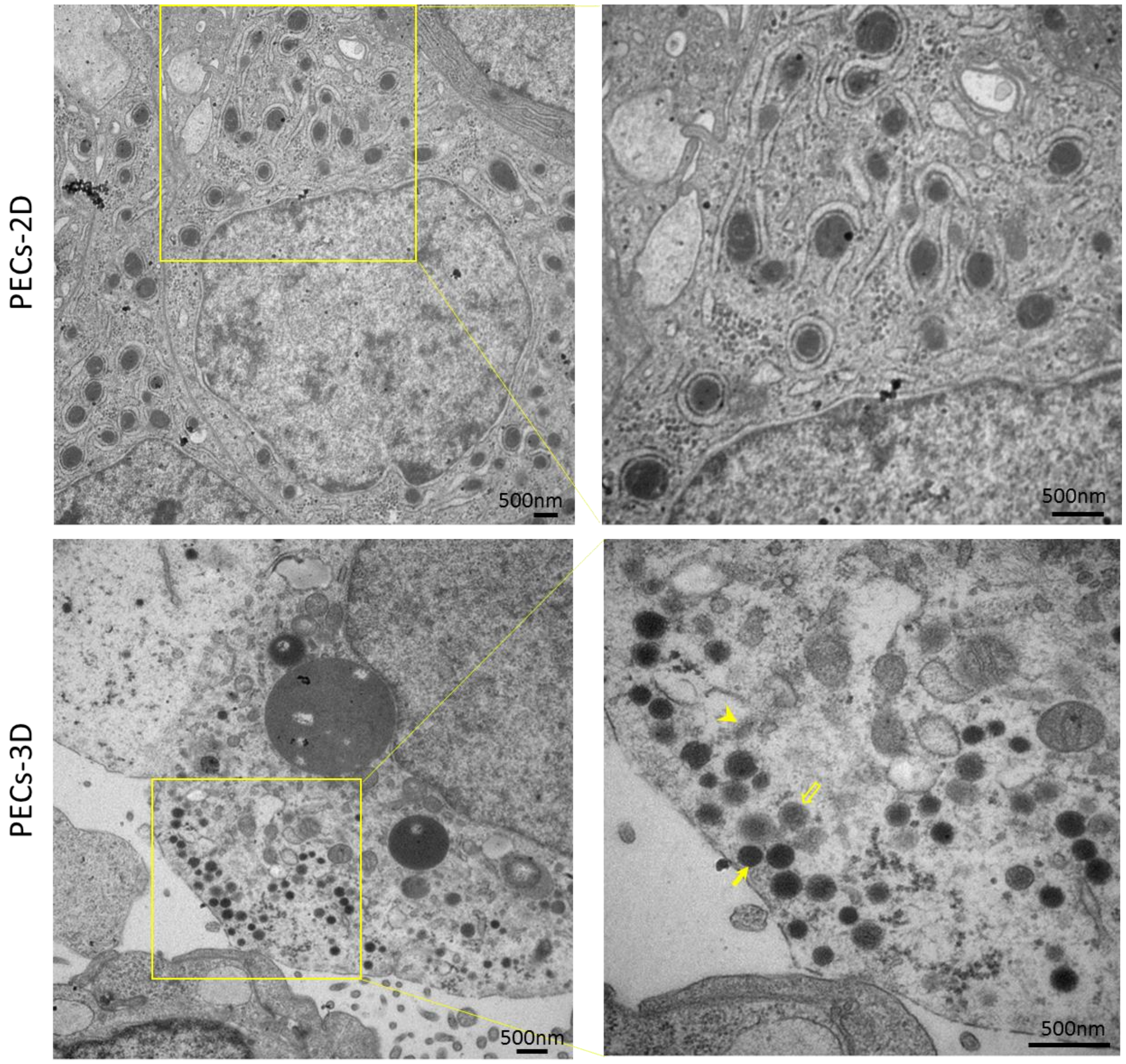

Figure S4. TEM images of PECs-2D or PECs-3D illustrating the ultrastructure of endocrine granules. Insulin granules could be categorized into three main types: pale, diffuse gray core (open arrow); dense round core (solid arrow); dense rod-shaped core (arrowhead). Scale bars, 500 nm.

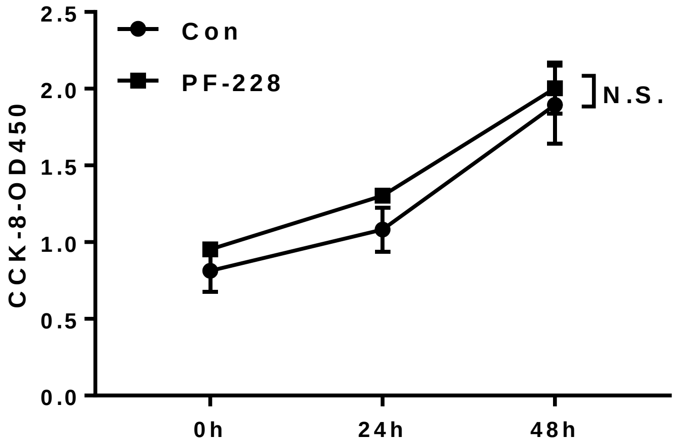

Figure S5. The proliferative ability of cells treated with or without PF-228, evaluated by CCK-8 assay. N.S., not significant.

Table S1. Primers used in this study.

**Gene**	**Forward**	**Reward**
GAPDH	GAGTCAACGGATTTGGTCGT	TTGATTTTGGAGGGATCTCG
SOX17	GTGGACCGCACGGAATTTG	GAGGCCCATCTCAGGCTTG
FOXA2	GCGACCCCAAGACCTACAG	GGTTCTGCCGGTAGAAGGG
HNF6	CAAACCCTGGAGCAAACTCAA	TGTGTTGCCTCTATCCTTCCC
PROX1	TTGACATTGGAGTGAAAAGGACG	TGCTCAGAACCTTGGGGATTC
PDX1	TTAGGATGTGGACGTAATT	GGTCAAGTTCAACATGACAG
NKX6.1	AGGACGACGACTACAATAAGCCT CTG	CGCTGCTGGACTTGTGCTTCT
NEUROG 3	GGAGTCGGCGAAAGAAGGC	TACAAGCTGTGGTCCGCTATG
INSULIN	GCAGCCTTTGTGAACCAACAC	CCCCGCACACTAGGTAGAGA
SST	GCTGCTGTCTGAACCCAAC	CGTTCTCGGGGTGCCATAG
AMY2A	TTCAGACCTTGGTGGGAAAGA	ACGAACCCCAACATTGTTACAT
GLUCAGO N	GACAAGCGCCATTCACAGG	TGACGTTTGGCAATGTTATTCCT
Cx36	GTGTACGATGATGAGCAGACC	GGAAGACCCAGTAACGTATGTG
ECAD	AGGAATTCTTGCTTTGCTAATTCT G	CGAAGAAACAGCAAGAGCAGC
PAX6	AGGTATTACGAGACTGGCTCC	TCCCGCTTATACTGGGCTATTT

Table S2. Antibodies used in this study.

**Primary Antibody**		
**Antibody**	**Company**	**Dilution**
NCAM(CD56)	BD	100(IF)
CXCR4	Abcam	500(IHC)<br>200(IF)
EpCAM	Neomarker	800(IHC)<br>200(IF, FACS)
Sox17	R&D Systems Inc	200(IHC)<br>100(IF, FACS)
FoxA2	R&D Systems Inc	200(IHC)<br>100(IF, FACS)
Somatostatin	Millipore	100(IF)
Insulin	Abcam	100(IF)
Glucagon	Sigma-Aldrich	200(IF)
c-peptide	Millipore	100(IF)
PDX1	Abcam	200(IF)

NKX6.1	R&D	200(IF)
Cx36	Abcam	500(IF, WB)
β-actin	Santa Cruz	1000(WB)
FAK-pY397	Upstate Biotechnology	1000(WB)
FAK	Upstate Biotechnology	1000(WB)
SMAD2	Cell Signaling Technology	1000(WB)
CD142-PE	BD	100(FACS)
**Secondary Antibody**		
**Secondary Antibody**	**Company**	**Dilution**
Alexa Fluor® 568 Goat Anti-Mouse IgG$_1$ (γ1)	Invitrogen	400
Alexa Fluor® 488 Goat Anti-Mouse IgG$_{2a}$ (γ2a)	Invitrogen	400
Alexa Fluor® 647 Goat Anti-Mouse IgG$_{2b}$ (γ2b)	Invitrogen	400
Alexa Fluor® 647 Goat Anti-Rabbit IgG (H+L)	Invitrogen	400
Alexa Fluor® 488 Goat Anti-Guinea Pig IgG (H+L)	Invitrogen	400
Alexa Fluor® 647 Goat Anti-Rat IgG (H+L)	Invitrogen	400
Alexa Fluor® 568 Goat Anti-Mouse IgG (H+L)	Invitrogen	400
Alexa Fluor® 647 Donkey Anti-Mouse IgG (H+L)	Invitrogen	400
Alexa Fluor® 568 Donkey Anti-Mouse IgG (H+L)	Invitrogen	400
Alexa Fluor® 488 Donkey Anti-Mouse IgG (H+L)	Invitrogen	400
Alexa Fluor® 568 Donkey Anti-Goat IgG (H+L)	Invitrogen	400
Alexa Fluor® 488 Donkey Anti-Rabbit IgG (H+L)	Invitrogen	400
Alexa Fluor® 568 Goat Anti-Mouse IgG$_{2a}$ (γ2a)	Invitrogen	400
